# Crystal structures of Triosephosphate Isomerases from *Taenia solium* and *Schistosoma mansoni* provide insights for vaccine rationale and drug design against helminth parasites

**DOI:** 10.1371/journal.pntd.0007815

**Published:** 2020-01-10

**Authors:** Pedro Jimenez-Sandoval, Eduardo Castro-Torres, Rogelio González-González, Corina Díaz-Quezada, Misraim Gurrola, Laura D. Camacho-Manriquez, Lucia Leyva-Navarro, Luis G. Brieba

**Affiliations:** Laboratorio Nacional de Genómica para la Biodiversidad, Centro de Investigación y de Estudios Avanzados del IPN, Irapuato, Guanajuato, México; Institut Pasteur de Montevideo, URUGUAY

## Abstract

Triosephosphate isomerases (TPIs) from *Taenia solium* (TsTPI) and *Schistosoma mansoni* (SmTPI) are potential vaccine and drug targets against cysticercosis and schistosomiasis, respectively. This is due to the dependence of parasitic helminths on glycolysis and because those proteins elicit an immune response, presumably due to their surface localization. Here we report the crystal structures of TsTPI and SmTPI in complex with 2-phosphoglyceric acid (2-PGA). Both TPIs fold into a dimeric (β-α)_8_ barrel in which the dimer interface consists of α-helices 2, 3, and 4, and swapping of loop 3. TPIs from parasitic helminths harbor a region of three amino acids knows as the SXD/E insert (S155 to E157 and S157 to D159 in TsTPI and SmTPI, respectively). This insert is located between α5 and β6 and is proposed to be the main TPI epitope. This region is part of a solvent-exposed 3_10_–helix that folds into a hook-like structure. The crystal structures of TsTPI and SmTPI predicted conformational epitopes that could be used for vaccine design. Surprisingly, the epitopes corresponding to the SXD/E inserts are not the ones with the greatest immunological potential. SmTPI, but not TsTPI, habors a sole solvent exposed cysteine (SmTPI-S230) and alterations in this residue decrease catalysis. The latter suggests that thiol-conjugating agents could be used to target SmTPI. In sum, the crystal structures of SmTPI and TsTPI are a blueprint for targeted schistosomiasis and cysticercosis drug and vaccine development.

## Author summary

Because of the worldwide prevalence of schistosomiasis and cysticercosis, it is critical to develop drugs and vaccines against their causative agents. The glycolytic enzyme triosephosphate isomerase (TPI) is a dual-edged sword against diseases caused by parasitic helminths. This is because helminths heavily depend on glycolysis for energy and because the surface localization exhibited by TPIs that elicits an immune response against those organisms. Here we provide the crystal structures TPIs from *Taenia solium* and *Schistosoma mansoni* as a first step for vaccine and drug design. As a proof of concept we found that modifications in the single solvent exposed cysteine of TPI from *S*. *mansoni* decreases catalysis, making this enzyme a novel target against schistosomiasis.

## Introduction

Helminths are parasitic worms responsible for diseases that collectively affect one-third of the human population [1–3]. Helminths are divided in two phyla: nematodes and platyhelminths. Nematodes include intestinal and filarial worms, whereas platyhelminths include flukes (trematodes) and tapeworms (cestodes) [4, 5]. Species from each phylum are associated with devastating human diseases. For instance, infection with the platyhelminth *Taenia solium*, may result in cysticercosis, a major cause of epilepsy in developing countries [6, 7] and nematodes from the genus *Schistosoma*, are the causative agents of schistosomiasis (snail fever or bilharzia) in humans [8]. Schistosomiasis in domesticated animals increases livestock morbidity and mortality resulting in economical losses specially in Asia and Africa [9].

Helminths depend on glycolysis for energy production, and several central metabolic enzymes from these parasites are candidates for drug design and vaccine development [10–12]. Among those enzymes, triosephosphate isomerase (TPI) is a widely studied target for rational drug design in protozoan parasites[13–19]. During glycolysis, TPI interconverts glyceraldehyde-3-phosphate and dihydroxyacetone phosphate with near diffusion-limited rates [20], a reaction that is necessary for energy production and to build precursors for the biosynthesis of amino acid and lipids and to prevent the accumulation of dihydroxyacetone phosphate that drives the accumulation of toxic methylglyoxal [21, 22]. TPIs display a (β/α)_8_ barrel or TIM-barrel fold and their active site consists of three invariable catalytic residues (Lys, His, and Glu)[23, 24]. Mutants that affect dimerization abrogate enzymatic activity, leading to the concept that TPIs are obligated dimers [25–28]. TPI activity is essential in amitochondriate parasites and in organisms, such as helminths or trypanosomatids, that heavily depend on glycolysis [29]. Besides their metabolic role, TPIs from other parasites like *Trichomonas vaginalis*, *Paracoccidioides brasiliensis*, and *Staphylococcus aureus* are involved in cell adhesion [30–32].

Upon infection, TPIs from helminths elicit an antibody response as this protein localizes on the surface of the parasite or is secreted [33–36]. TPI is a vaccine candidate against *S*. *japonicum* infection in mice, buffaloes, and pigs [10, 11, 34, 37, 38]. Furthermore, a chimeric vaccine based on the TPI and the heat shock factor 70 protein of *S*. *japonicum* significantly reduced the infection symptoms in animals [38]. Antibodies prepared against TPsI from *T*. *solium* and *S*. *mansoni* inhibit their catalytic activities [39–41]. These results suggest that TPI is potential component as a vaccine candidate against cysticercosis and schistosomiasis.

Phylogenetic analysis indicates that TPIs from parasitic flatworms harbor a three amino acids motif (SXD/E) not present in TPIs from non-parasitic flatworms or TPIs from the hosts. This region is a putative target to design vaccines or drugs against schistosomiasis and cysticercosis [42]. Although triosephosphate isomerases are a possible target for vaccine and drug design against helminth associated diseases, the only structural information of a triosephosphate isomerase from a helminth is the one from the trematode *Opisthorchis viverrini* (OvTPI) [43]. Here we determined the crystal structures of TPIs from *Taenia solium* (TsTPI) and *Schistosoma mansoni* (TsTPI) in complex with their inhibitor 2-phosphoglyceric acid (2-PGA) to assess whether those structures could be used as direct scaffolds against cysticercosis and schistosomiasis.

## Methods

### TsTPI and SmTPI subcloning and protein purification

The nucleotide coding sequences of TPI from *T*. *solium* (TsTPI) and *S*. *mansoni* (SmTPI) (GenBank: AAG21132.1 and XP_018647623 respectively) [44, 45] were codon optimized and synthetically synthesized for their expression in *E*. *coli* **(S1 Table).** The synthetic genes were subcloned into the *Nde* I and *Bam* HI restriction sites of a modified pET19 vector. Both proteins were expressed in an *E*. *coli* strain devoid of its endogenous triosephosphate isomerase gene [46] and purified following the protocol for *Trichomonas vaginalis* TPIs [47]. Recombinant TPIs have three additional amino acids (Gly, Pro, and His) before their initial N-terminal methionine. Proteins were stored in a buffer containing 100 mM TEA pH 7.4, 50 mM NaCl, 2mM DTT, and 1mM EDTA at 4°C for no more than two weeks. TPIs were reduced previously to all biochemical assays with 20 mM dithiothreitol (DTT) for 1 h at room temperature. Excess DTT was removed by using a prepacked Sephadex G-25 column.

### Enzyme kinetics and *in vivo* complementation assays

Catalytic activity was measured in the direction of glyceraldehyde 3-phosphate (G3P) to dihydroxyacetone phosphate (DHAP) by a coupled enzymatic assay assisted by α-glycerophosphate dehydrogenase (α-GDH) [48]. Assays were performed in 0.1 M TEA-HCl pH 7.4, 10 mM EDTA, 0.2 mM NADH, 1 μg α-GDH, and 1 mM of D-L glyceraldehyde 3-phosphate. Enzymatic reactions started by addition of SyTPI or TsTPI and enzymatic activities were calculated by the decrease in absorbance at 340 nm at 21 ºC. The determination of the kinetic constants, K_M_ and k_cat_, was performed varying G3P concentrations from 0 to 5 mM. *In vivo* complementation assays were conducted on a Δ*tpi* BL21 *DE3 E*. *coli* strain as previously described [46, 47].

### Protein crystallography and structural determination

Concentrated TsTPI and SmTPI (20 mg/ml) were incubated with 10 mM of 2-phosphoglyceric acid (2-PGA) for 30 minutes on ice in protein storage buffer. Crystallization trails were assayed employing the sitting drop method using 1 μl of protein-inhibitor complex and 1 μl of the reservoir solution. Pyramidal crystals of TsTPI appeared after 2 days in a reservoir solution containing 0.04 M potassium phosphate monobasic and 16% w/v polyethylene glycol 8,000, whereas SmTPI crystals appeared overnight in a solution containing 0.05 M cesium chloride, 0.1 M MES monohydrate pH 6.5, and 30% v/v Jeffamine M-600. TsTPI crystals were dipped into a cryo-protectant solution containing 20% of glycerol, whereas SmTPI crystals were directly taken from the crystallization drop and both flash-frozen in liquid nitrogen. Diffraction was collected on a Micromax 002+ diffractometer (Rigaku) equipped with a sealed tube conventional X-ray source. A single dataset was integrated and scaled using XDS and XSCALE respectively [30]. Phases were solved by molecular replacement using the program PHASER [31] and the crystal structure of TPI from *Litopenaeus vannamei* [49] as a search model. Initial model and refinement were executed using COOT and PHENIX. Structural coordinates were deposited with PDB accession numbers 6OOG and 6OOI, for TsTPI and SmTPI, respectively (**S2 Table**).

### Fluorescence-based thermal-shift assay (TSA)

TSA was performed on a real time PCR device (Step One Instrument 48 wells, Applied Biosystems), accordingly to published protocols [50]. Briefly, purified proteins were diluted in 25 mM Tris buffer pH 8.0, 100 mM NaCl to a final concentration of 4 μM. Nine microliters of 4 μM protein solution were mixed with 1 μL of Sypro Orange dye 20X. The final concentration of Sypro Orange dye in the sample was 2X and the final volume was 10 μL. Excitation was at 490 nm and fluorescence was recorded at 575 nm. The melting curve was set from 25 to 95°C, increasing the temperature by 1°C each two minutes. Data were analyzed using the Protein Thermal Shift software from Applied Biosystems. Assays, performed in triplicate.

### 2-nitro-5-thiocyanobenzoic acid (NTCB) cysteine footprinting

NTCB cleavage was performed as previously described [51]. Purified proteins were incubated for 1 hour at 37°C in reduction buffer (50 mM Tris-HCl pH 8.0, 100 mM NaCl and 5 mM DTT) to remove disulfide bonds. Reduced proteins were dialyzed against 50 mM Tris-HCl pH 8.0 and 100 mM NaCl to remove DTT. A 10-fold molar excess of NTCB over protein cysteine content was added to each sample. After incubation of 2 hours at 20°C proteins were dialyzed against sample buffer to remove non-reacted NTCB. For denaturation, proteins were buffer exchanged into unfolding buffer (50 mM Tris-HCl pH 8.0, 100 mM NaCl and 5 M urea). Cleavage was initiated by raising the pH of the sample to pH 9.0 with 1 M NaOH. The reactions were incubated overnight at 37°C, stopped by addition of 3 mM β-mercaptoethanol and analyzed by SDS-PAGE.

### Spectrophotometric determination of reactive thiols

The number of free thiols of SyTPI and TsTPI were determined spectrophotometrically with 5,5′-Dithiobis(2-nitrobenzoic acid) (DTNB) [52]. For this assay 90 μL of previously quantified protein was added to a solution containing 200 μM of DTNB (SIGMA, USA) in 100 mM Na_2_PO_4_ pH 8.0. Absorbance at 412 nm was determined after 5 min. of incubation at room temperature. A L-cysteine calibration curve was used to calculate the concentration of titrated sulfhydryl groups.

### Site-directed mutagenesis

Residue TsTPI-C222 was mutated to Asp (D), Tyr (Y), Lys(K), and Ser (S) by site directed mutagenesis using the Q5 protocol from New England Biolabs and confirmed by Sanger sequencing.

## Results

### Multiple Sequence Analysis and Purification of TsTPI and SmTPI

**Fig 1:**
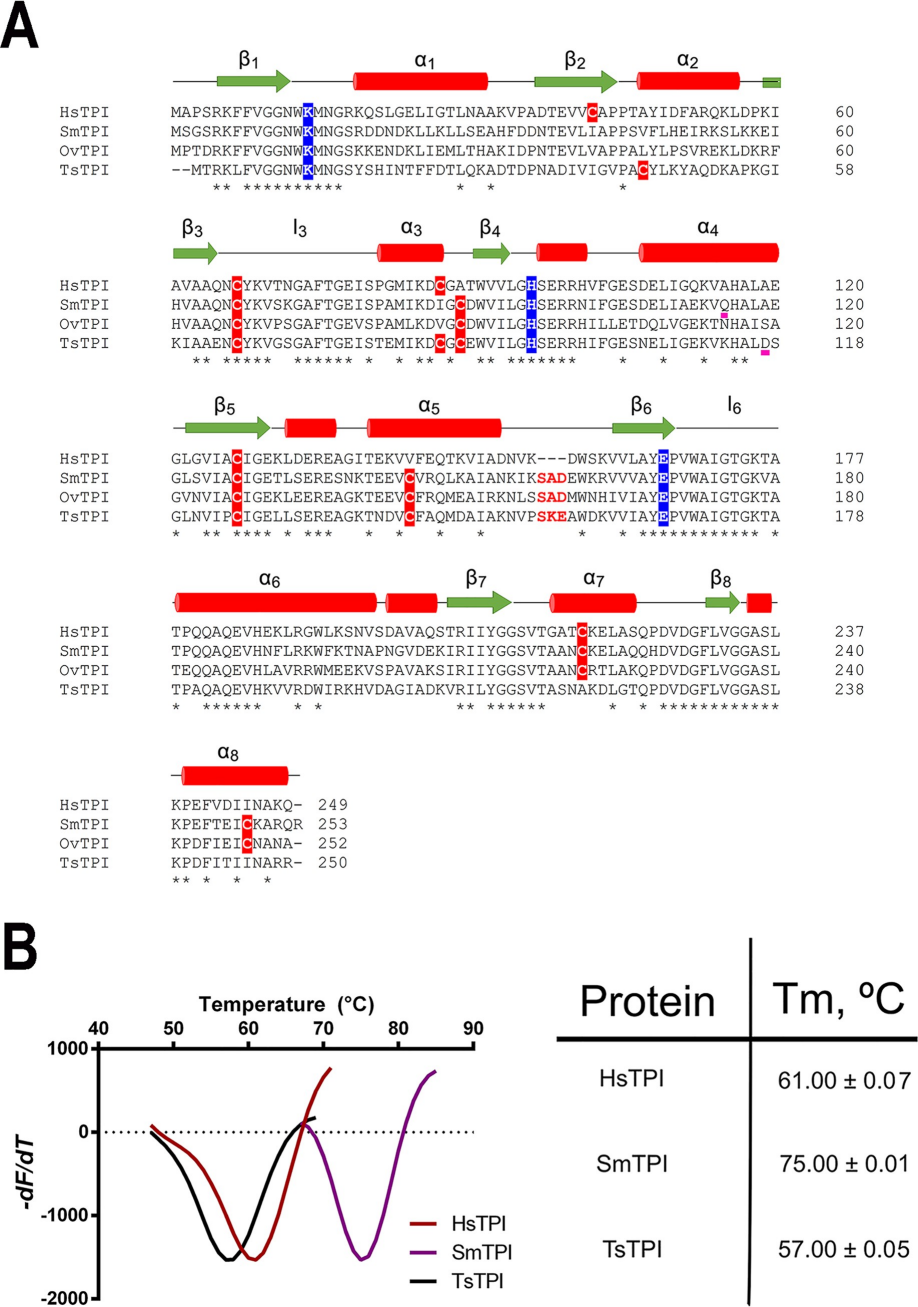
Structural alignment and thermal stability of helminths and human TPIs. **A)** Amino acid sequence alignment of TPIs from *T*. *solium*, *S*. *mansoni*, *O*. *viverrini*, and *H*. *sapiens*. The three invariable catalytic amino acids are colored in blue. Residues K12, H94, E167 correspond to *T*. *solium* and K14, H96, E169 are from *S*. *mansoni*. Cysteine residues and the SXD/E motif are colored in red. **B)** Thermal unfolding curves of TPIs from *T*. *solium*, *S*. *mansoni*, and *H*. *sapiens* measured by a fluorescence-based thermal-shift assay. The curves show the first derivative of the changes in fluorescence. For these data, the Tm is calculated as the inflection point in the curve. Data were produced in triplicate.

TPIs exhibit a similar length in their secondary structural elements and are not prone to insertions or deletions [53]. TsTPI and SmTPI share 59% amino acid identity and a distinctive feature is the presence of a region of three amino acids, (S155 to E157 and S157 to D159, in TsTPI and SmTPI, respectively) conserved among flatworms that is not present in HsTPI and non-parasitic helminths [42]. **(Fig 1A).** Both TsTPI and SmTPI contain six cysteines that could serve as targets for rational drug design using thiol-conjugating agents.

### Kinetic properties and biophysical characterization of TsTPI and SmTPI

SmTPI and TsTPI were purified to homogeneity with a yield of approximately 5 mg/ml after two purification steps (**S1 Fig**). Steady-state kinetics of recombinant TsTPI and SmTPI show that those enzymes present similar kinetic profiles to those previously reported and with the activity ranges of TPIs from *Fasciola hepatica* and *Brugia malayi* [33, 44, 45, 54, 55] (**Table 1**).

**Table 1:** Steady-state kinetic parameters of recombinant TsTPI and SmTPI in comparison to HsTPI.

Enzyme	K_m_(mM)	k_cat_(s^-1^)	k_cat_/K_m_(M^-1^ s^-1^)	Fold decrease
HsTPI	0.61 ± 0.02	4010 ±	6.54x10^6^	-
TsTPI	0.31 ± 0.03	993 ± 44.0	3.15x10^6^	-
SmTPI	0.48 ± 0.07	2872 ± 138	5.80x10^6^	-
SmTPI-C221S	0.87 ± 0.12	2494 ± 196	2.84x10^6^	2.04
SmTPI-C221K	2.57 ± 0.93	1396 ± 312	5.41x10^5^	10.7
SmTPI-C221Y	1.60 ± 0.50	875 ± 145	5.45x10^5^	10.6
SmTPI-C221D	2.84 ± 0.88	1215 ± 205	4.27x10^5^	13.5

SmTPI has a melting temperature of 82 ºC and *Fasciola hepatica* TPI (FhTPI) of 67 ºC [45, 54]. To ask if these high melting temperatures are a common feature in TPIs from helminths, we measured the melting point of TsTPI and SmTPI. Our analysis indicates that SmTPI and TsTPI exhibit a Tm of 75 and 57 ºC, respectively. **(Fig 1B**). The thermal stabilities observed by FhTPI and TsTPI are similar to the melting temperature exhibited by HsTPI and yeast TPI (ScTPI), that are 66.2 and 63°C, respectively [56] (**Table 2**). Thus, the high melting temperature displayed by SmTPI is not conserved among TPIs from other helminths that exhibit similar melting temperatures to TPIs from other mesophilic organisms.

**Table 2:** Melting temperatures of Wild-type and mutant TPIs.

Enzyme	Tm,°C
HsTPI	61.0 ± 0.7
TsTPI	57.0 ± 0.5
SmTPI	75.0 ± 0.1
SmTPI-C221S	74.5 ± 0.3
SmTPI-C221K	69.8 ± 0.2
SmTPI-C221Y	70.7 ± 0.4
SmTPI-C221D	69.1 ± 0.6

### Crystal structures of TsTPI and SmTPI

**Fig 2:**
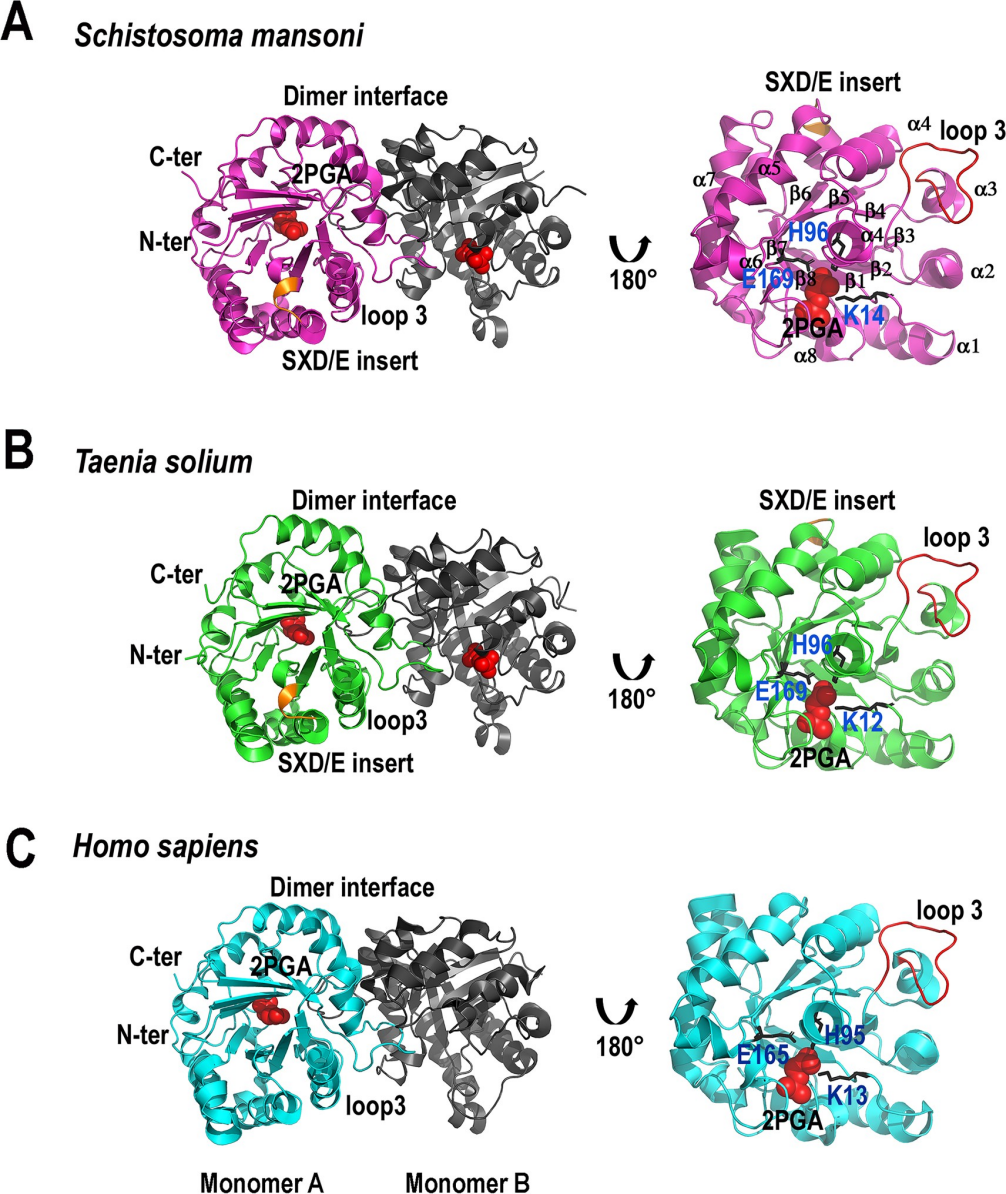
Crystal structures of TsTPI and SmTPI in comparison to HsTPI. **A)** Ribbon representation of SmTPI. Monomer A is colored in magenta and monomer B is colored in black. Residues corresponding to the SXD/E motif are colored in orange. The 2-PGA inhibitor is colored in red and represented as spheres. **B)** Ribbon representation of SmTPI. Monomer A is colored in green and monomer B in black. The SXD/E motif is also colored in orange. **C)** Ribbon representation of HsTPI. Monomer A is colored in blue. In this crystal structure the inhibitor 2-PGA is bound in only one subunit [59]. The right part of the figure shows a magnification of monomer A for SmTPI, TsTPI, and HsTPI. Loop 3, involved in the assembly the dimer interface, is colored in red. The conserved structural secondary elements are labeled only for SmTPI.

We solved the crystal structures of TsTPI and SmTPI in complex with the inhibitor 2-PGA at 2.1 and 2.3 Å, respectively (**Fig 2**). TsTPI crystallized as one monomer per asymmetric unit that forms a biological dimer with a symmetry-related molecule, whereas SmTPI contained 8 monomers (or 4 dimers) in its asymmetric unit. The electron density for all 250 and 253 amino acids of TsTPI and SmTPI, respectively, are visible in their electron density maps and the 2-PGA inhibitor is bound in all monomers. The all-atom rmsd (root mean square deviation) between both TsTPI and SmTPI is 0.572 Å. TPIs are dimeric enzymes with a large buried accessible area. The total accessible area in the dimeric SmTPI and TsTPIs as dimers is 18,610 Å^2^ and 19,402Å^2^, respectively [57]. SmTPI and TsTPI display dimer surface interface areas of 1567 and 1018 Å^2^ per monomer, respectively, that compares with the surface interface area of 1681 Å^2^ of HsTPI [58]. As in other TPIs, the dimer interactions are mainly held by inter-subunit contacts between loop 3 of one subunit with a hydrophobic surface from the other subunit (**Fig 2A and 2B**). In TIM-barrels, the loops located after the β-strands assemble the front or catalytic part of the barrel and the loops situated after the α-helices assemble the posterior or structural part of the barrel. In the front part of the barrel, the essential catalytic amino acids acid of TsTPI (K12, H94, E167) and SmTPI (K14, H96, E169) are positioned to directly interact with the 2-PGA inhibitor (**Fig 2A and 2B**). The SXD/E insert is located in the posterior part of the barrel between α5 and β6. In both SmTPI and TsTPI the SXD/E insert folds as a 3_10_-helix that connects α5 and β6, whereas this secondary structural element is absent in other TPIs like HsTPI (**Fig 2C**)

### Closed conformation of TsTPI and SmTPI

**Fig 3:**
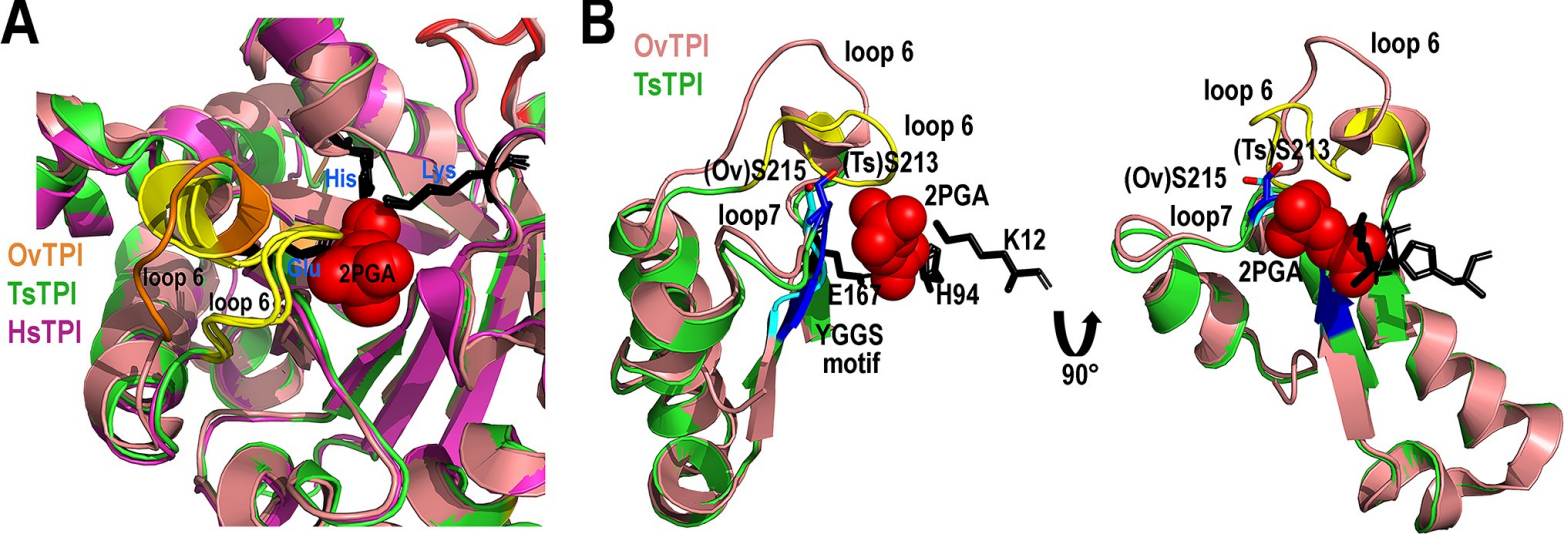
Closed and open conformations of TPIs from helminths. **A)** Close-up view of the active site of TsTPI and SmTPI in comparison to OvTPI. Structural alignments of TPIs from helminths solved in the presence of 2-PGA (TsTPI and SmTPI) and in its absence (OvTPI). The loop 6 and β-stand 7 of TsTPI and SmTPI are colored in yellow, whereas in OvTPI these structural elements are colored in orange. The conformational change in loop 6 results from a hinge motion in the presence of substrate. **B)** Substrate binding assembles the active site in helminth TPIs. Ribbon representation of residues corresponding to loops 6 and 7 in OvTPI and TsTPI. The figure illustrates the positions of residues TsTPI-S213 and SmTPI-S215. The position of the conserved YGGS motif of the phosphate binding loop are colored in blue and cyan. The active site amino acids of TsTPI are in a ball-stick representation and black colored.

Crystal structures of TPIs in the presence and absence of ligands, have shown that in the absence of substrate analogs TPIs adopt an “open” conformation, whereas in the presence of ligands, TPIs exhibits a “closed” conformation in which loop 6 moves towards the active site [60–62]. Loop 6 is a highly dynamic structural element that alternates between different conformers. A structural superposition of TsTPI and SmTPI (crystallized in the presence of 2PGA), with the crystal structure of TPI from the parasitic helminth *Opisthorchis viverrini* (OvTPI) (crystallized in the absence of substrate [43]), shows that the main differences between these structures is related to the orientation of loop 6 (**Fig 3A**). In TsTPI and SmTPI, loop 6 adopts a “closed” conformation necessary for catalysis. The closed conformation is recognized by a 7.5 Å displacement of residue G175 at the tip of loop 6 in TsTPI with respect to the corresponding residue in OvTPI (G177) (**Fig 3B**). No crystallographic contacts are observed in residues near loop 6 in TsTPI and SmTPI, supporting the role of substrate binding in promoting the closed conformation. The universally conserved catalytic residues Lys, His, and Glu are located in identical positions in TsTPI and SmTPI and are in an optimal position to interact with the substrate analog (**Fig 3A**). As in other TPIs, residues from loop 6 interact with the conserved tyrosine and serine residues from the YGGS motif of loop 7 via hydrophobic and hydrogen bond interactions (**Fig 3B**) [63, 64]. Accordingly, the hydroxyl group of Ser 213 in TsTPI and SmTPI interacts with the substrate analog, whereas in OvTPI the hydroxyl group of this residue moves away from the substrate (**Fig 3B**). The crystal structures of helminthic TPIs are consistent with the role of loops 6 and 7 to modulate structural rearrangements necessary for substrate binding and catalysis.

### Structural comparison of the SXD/E insert

**Fig 4:**
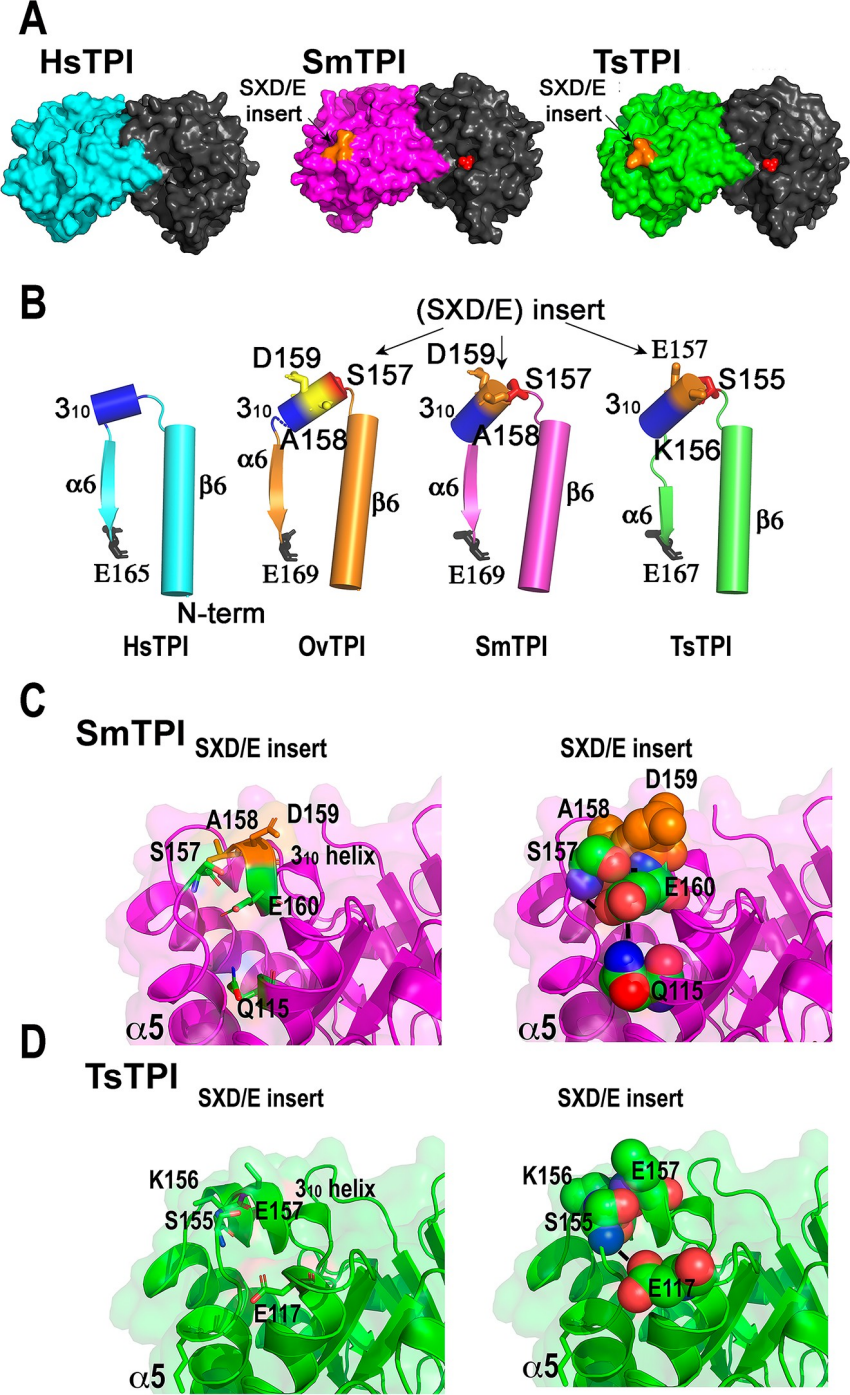
Structural localization of the SXD/E insert in TPIs from helminths. **A)** Surface representation of human and helminth TPIs focusing on the immunogenic SXD/E insert. The hook-like structure that adopts the SXD/E insert in both SmTPI and TsTPI is colored in orange **B)** Ribbon representation of the α6, 3_10_-helix, and β6 in representative TPIs. The SXD/E insert of SmTPI and TsTPI are colored in orange and in yellow for OvTPI. **C and D**) Close view of the hydrogen bond interactions that stabilize the SXD/E insert in SmTPI and TsTPI.

The three amino acid SXD/E insert between β6 and α6 generates a distinctive solvent surface area in those enzymes (**Fig 4A**). In HsTPI and non-parasitic helminths β6 and α6 are connected by a short 3_10_-helix (D156-K159) (**Fig 4B**) [42]. In TPIs from helminths this 3_10_ helix consists of seven amino acids (residues S155 to K161 in TsTPI; S157 to R163 in SmTPI and S157 to H163 in OvTPI) (**Fig 4B**). The character of the serine and acidic amino acids (aspartate or glutamate) is conserved. Both SmTPI and OvTPI harbor an alanine in the middle of the SXD/E insert, whereas TsTPI harbors a lysine (**Figs 1A and 4B**). In SmTPI, the 3_10_-helix is stabilized via hydrogen bond interactions between residue E160 with residues S157 and Q115, and in TsTPI residue S155 forms a hydrogen bond with residue D117 (**Fig 4C and 4D**). These interactions contrast with the hydrogen bond interactions between residues S157 and Q115 in OvTPI [43]. In SmTPI, residue E160_,_ located one amino acid after the SAD insert, forms a hydrogen bond with residue Q115. This amino acid mediates a hydrogen bond network between the SXD/E insert and αhelix 4. In TsTPI residue S157 forms a hydrogen bond interaction with residue D117 that is located two amino acids after the analogous Q115 residue of OvTPI (**Fig 4C and 4D**).

### Conformational epitope prediction of TsTPI and SmTPI

**Fig 5:**
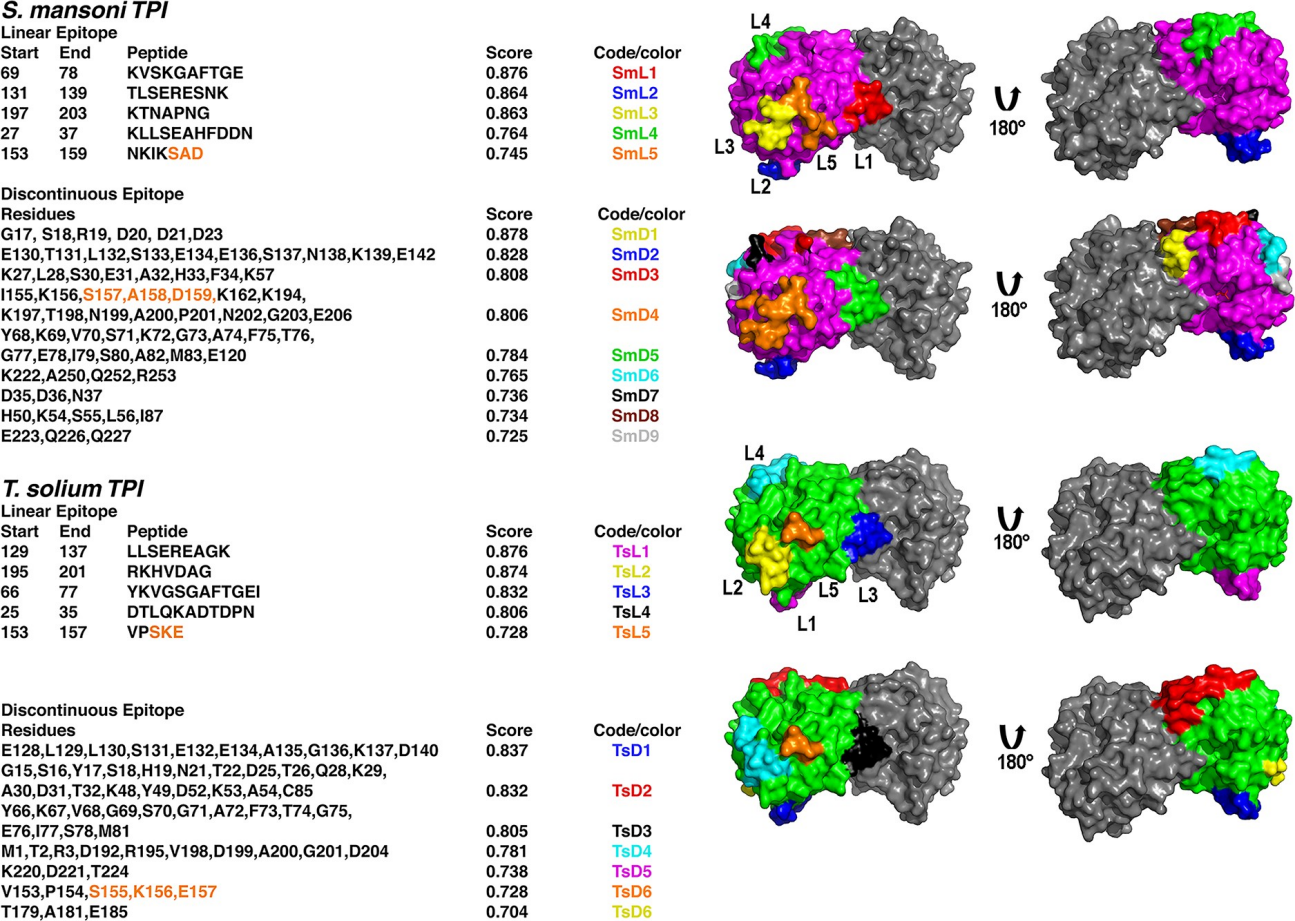
Prediction of linear and discontinuous epitopes in SmTPI and TsTPIs. The amino acid corresponding to the linear epitopes are depicted by their start and end, whereas the amino acids that compose the discontinuous epitopes are indicated using a single letter code. The probability score and the color code of epitope is indicated. The localization of individual epitopes is present only in molecule A of SmTPI and TsTPI accordingly to their code. The predicted epitopes harboring the SXD/E insert are colored in orange.

We used the program ElliPro [65] to interrogate the crystal structures of TsTPI and SmTPI for linear and conformational epitopes **(Fig 5)**. A total of 5 linear epitopes with a score greater than 0.7 are predicted to be present in TsTPI and SmTPI. Those 5 linear epitopes are conserved between TsTPI and SmTPI, although their indicators as probable elicitors of an immune response are not the same **(Fig 5)**. The linear epitopes include: 1) a segment of loop 3 (residues 65 to 78 in SmTPI and 66 to 77 in TsTPI), 2) a 3_10_ helix that connects β5 with α5 (residues 131 to 139 in SmTPI and 129 to 137 in TsTPI), 3) an α3_10_ helix located between α6 and β7 (residues 197 to 203 in SmTPI and 195 to 201 in TsTPI) 4) the C-terminal of α1 and the loop that connect this α-helix with β2 (residues 27 to 37 in SmTPI and 25 to 35 in TsTPI) and 5) the α3_10_ helix that harbors the SXD/E inserts, 153 to 159 in SmTPI and 153 to 157 in TsTPI **(Fig 5)**. The epitope with the lowest score predicted to elicit immune response corresponds to the SXD/E inserts in both SmTPI and TsTPI (SmL5 and TsL5). A structural analysis using the crystal structure of HsTPI in complex with 2-PGA also produced 5 linear epitopes with a score higher than 0.7 (**S2 Fig**). From those 5 epitopes, only one epitope is conserved between HsTPI and TPIs from *S*. *mansoni* and *T*. *solium*. This epitope corresponds to the C-terminal part of α1 and the loop that connects this α-helix with β2 (residues 26 to 36 in HsTPI) (**S1 Fig**). This observation suggests that linear epitopes STPI_L1,_ STPI_L2,_ STPI_L3_ and TsTPI_L1,_ TsTPI_L2,_ TsTPI_L3_ could be used in combination with the previously characterized SXD/E derived epitopes (SmL5 and TsL5) to elicit an immune response against *T*. *solium* and *S*. *mansoni*.

### Structural rational for SmTPI inhibition by thiol conjugating agents

**Fig 6:**
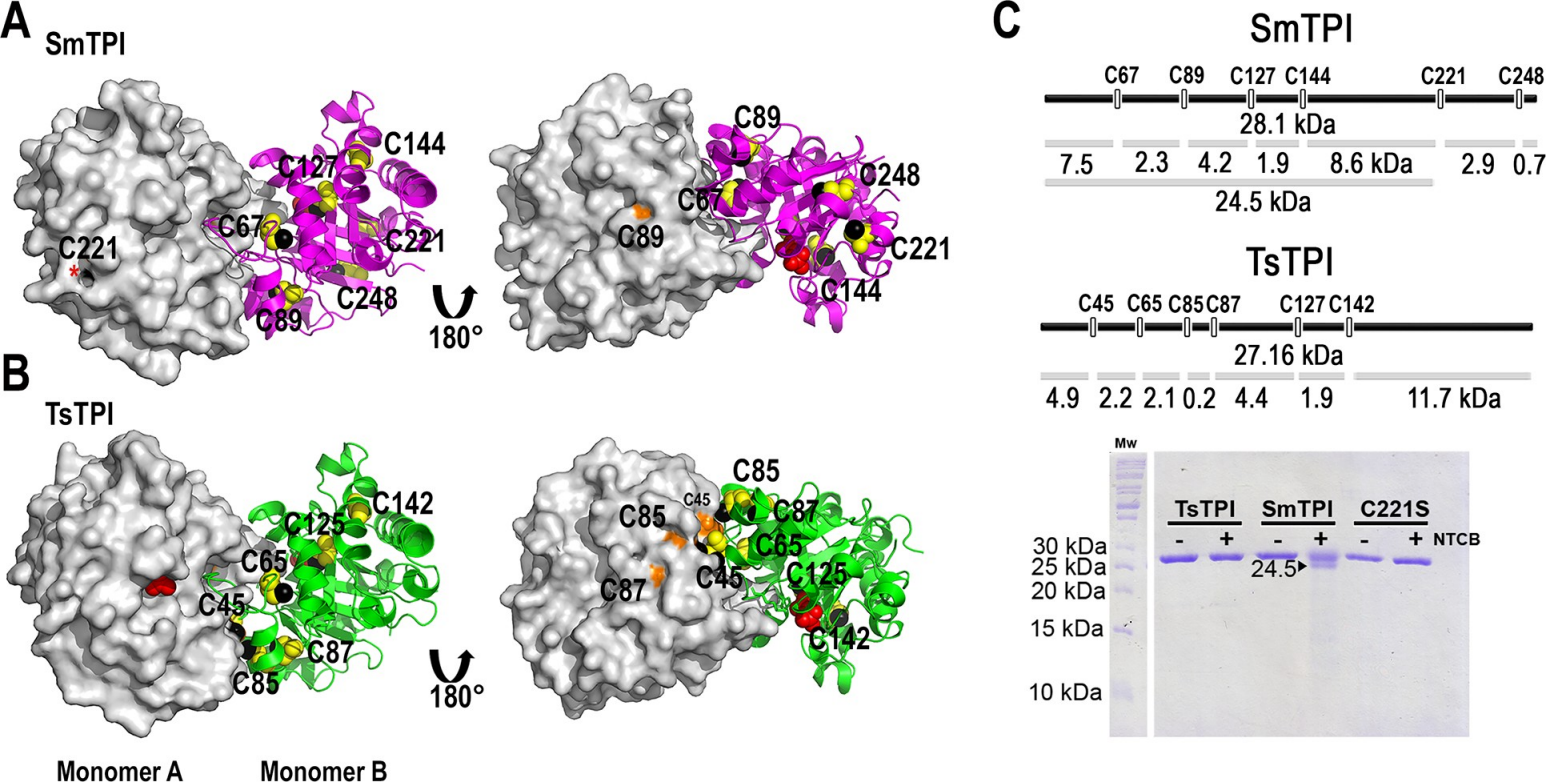
Crystal structure of SmTPI and TsTPI showing their cysteine residues. **A and B)** Surface and ribbon representation of SmTPI and TsTPI showing their exposed cysteines. Molecule A in both TPIs is presented as a surface representation, whereas molecule B is show as ribbons. The thiol group of each cysteine is colored in black in both molecules A and B. The rest of the atoms of the cysteines are colored in orange for molecule A and yellow for molecule B, respectively. The thiol group of residue SmTPI-C221 is labeled with a red asterisk. **C)** Predicted and experimental NTCB cleavage sites in SmTPI and TsTPI. The upper part of the panel shows the predicted NTCB cleavage sites for SmTPI and TsTPI, whereas the bottom part of the panel shows a SDS-gel with protein samples treated with NTCB. SmTPI is processed by NTCB at a single site generating a product of 24.5 kDa and this product is not present in the SmTPI-C221S mutant.

TsTPI and SmTPI harbor 6 cysteine residues in their primary sequence (**Fig 1A**). Accordingly, to the crystal structures of TsTPI and SmTPI, the only thiol group that is partially solvent exposed in TsTPI and SmTPI is the one from residue C221 from SmTPI (**Fig 6A**). Although residues SmTPI-C89, TsTPI-C85, TsTPI-C87, and TsTPI-C45 have their Cβ side chain or main chain exposed to the solvent, their thiol groups are completely buried (**Fig 6A and 6B**). The number of cysteines harboring thiol accessible groups in TsTPI and SmTPI was determined by the use of 5, 5′-dithiobis (2-nitrobenzoic acid) (DTNB) and 2-nitro-5-thiocyanobenzoic acid (NTCB) footprinting. Accordingly, to the DTNB reaction, 0.52 and 1.02 accessible cysteines are present in TsTPI and SmTPI, respectively (**Table 3**).

**Table 3:** Accessible thiol per TPI monomer in wild-type and mutant TPIs.

Enzyme	Accesible thiols/monomer
TsTPI	0.52
SmTPI	1.02
Cys221Ser	0.49
HsTPI	0.98

Similarly, TsTPI is not cleaved by a NTCB reaction, whereas the addition of NTCB to SmTPI produces a single proteolytic cut (**Fig 6C**). The fractional number of accessible thiols per monomer determined by DTNB in TsTPI may be a consequence of the intrinsic flexibility of this enzyme that transiently exposes to the solvent the buried thiol groups. The migration pattern of the proteolytic product of SmTPI suggests that NTCB reacts and cuts near residue C221. In order to corroborate that residue SmTPI-C221 is the sole solvent exposed cysteine in SmTPI, we mutated this residue to serine and performed a NTCB cleavage assay (**Fig 6C**). The mutation SmTPI-C221S is not cleaved by NTCB, corroborating that the only accessible thiol in SmTPI is located at residue C221 (**Fig 6C**).

### Mutations in residue TsTPI C221 that mimic oxidative stress or thiol conjugating agents decrease enzymatic activity

Residue SmTPI-C221 is located at the N-terminus of α7, just a few amino acids after the conserved YGGS motif (residues 212 to 215). In order to investigate if modifications in SmTPI-C221 may impinge catalysis, we mutated this residue to Asp, Tyr, and Lys that mimic chemical modifications of a cysteine **(S3 Fig).** We mutated residue SmTPI-C221 to Asp that mimics the cysteine oxidation to sulfinic acid, to Tyr that mimics cysteine derivatization with an aromatic agent like DTNB and to Lys that mimics cysteine derivatization with a linear adduct like MMTS. A comparison of the catalytic parameters of the point mutants in residue SmTPI-C221 highlights that a conserved mutation to serine only decreases the catalytic efficiency by 2-fold, whereas mutations that mimic the conjugation or oxidation of residue SmTPI-C221 reduce their catalytic efficiency between 10.6 to 13.5-fold (**Table 1**). The introduction of these point mutants does not alter the melting point or the dimeric nature of those enzymes **(Table 2 and S4 Fig)**, suggesting that the decrease in enzymatic activity is due to conformational changes that alter catalysis and not by inducing SmTPI monomerization. We conducted a qualitative analysis using an *E*. *coli* strain devoid of *tpi* by plasmids harboring mutations in residue SmTPI-C221 **(S3 Fig)**. Only a few colonies are observed in bacterial cells harboring the less catalytically efficient mutant SmTPI-C221D, bacteria complement with mutants SmTPI-C221K and SmTPI-C221Y exhibited less number of colonies and bacteria complemented with SmTPI-C221S presented similar colonies than wild-type SmTPI-C221.

## Discussion

Helminth parasites are causal agents of diseases prevalent in humans. The only structural information of a TPI from a helminth is the one form the parasite trematode *Opisthorchis viverrini* [43], limiting the potential use of this crystal structure to opisthorchiasis and not necessary to other helminths. In order to have a structural scaffold to guide rational drug and vaccine design against parasitic helminths, we solved the crystal structures of TPIs from *T*. *solium* (TsTPI) and *S*. *mansoni* (SmTPI).

The crystal structures of TsTPI and SmTPI present an archetypical (β- α)_8_ or TIM-barrel fold composed of 8 alternate β-strand and α-helices [23]. SmTPI is 18°C more thermostable than TsTPI. Although these proteins are 60% identical their differences in amino acid composition are located in α1, β2, and α3. Within these secondary elements, residues R19 and E51 exert a salt bridge, residue K57 interacts with the carbonyl group of A32, and residue F34 is located in a hydrophobic cleft. The corresponding amino acids in TsTPI do not form a salt bridge or hydrogen bond that may contribute to stability (**S5 Fig**). The sum of the stabilizing interactions in SmTPI correlates with its higher melting point.

The most prominent characteristic of TPIs from parasitic helminths is the presence of a three amino acids insert (SXD/E) located between β6 and α6, that is not present in TPIs from non-parasitic helminths. Our crystal structures reveal that this motif folds into a 3_10_-helix that is solvent exposed in both TsTPI and SmTPI and that the addition of the SXD/E insertion creates a “hook-like structure” in both TPIs. The solvent exposure localization of the SXD/E insert creates a surface recognition zone that associates with the immune response associated with this motif. The hook-like structure in TsTPI and SmTPI is stabilized by a hydrogen bond interaction network.

Several groups have used recombinantly expressed TPIs as putative vaccines against schistosomiasis and cysticercosis [8, 9, 13, 14, 27, 46]. The precise determinants for immunogenic response in proteins are unknown, however factors like solvent accessibility, hydrophobicity, and flexibility are common features of an epitope. The crystal structures of TsTPI and SmTPI highlight the presence of four linear epitopes that are predicted to elicit a greater immune response than the one associated with the SXD/E insert. Relevantly, from those four inserts, only one is conserved in HsTPI. Thus, the crystal structures of TsTPI and SmTPI predict three new linear epitopes in each TPI that could be used to generate antibodies against *T*. *solium* and *S*. *mansoni*.

The prediction that the SXD/E insert elicits a weak immune response and its conservation among parasitic helminth are counterintuitive. TPIs from parasitic helminths are secreted proteins [33, 35, 36, 66] and the solvent exposed localization of the SXD/E insert suggests that this motif may be involved in the moonlighting proteins of parasitic TPIs [33] and thus a key component during helminth infection.

Specific derivatization of cysteines in TPIs from protozoan parasites result in the complete inactivation of these enzymes, making this approach a potential mechanism to design specific thiol-derivatizing agents. TPI has been a target for the design of specific inhibitors in several human parasites like *Trypanosoma cruzi*, *Giardia lambia*, and *Trichomonas vaginalis* [17, 67, 68]. Those inhibitors use the subtle differences in three-dimensional structures between TPIs from the host and the parasite to design inhibitors that are specific against the parasite´s TPI but are unreactive to the host. TPI inhibitors are divided into two types, those that are aimed to disrupt the dimer interface and those aimed at exposed cysteines to react with thiol conjugating agents [32–38]. Our data shown that none of the six cysteines in TsTPI harbors an accessible thiol group, whereas only one thiol group is accessible in SmTPI at position C221. Seminal studies demonstrate that in GlTPI thiol conjugating agents react with residue GlTPI-C222 via a disulfide bridge [29, 35, 39]. Residue GlTPI-C222 is structurally analogous to SmTPI-C221 suggesting the possibility that SmTPI may be inhibited by targeting its solvent accessible cysteine. Residues SmTPI-C221 and GlTPI-C222 localize near the YGGS motif that is necessary to assemble the closed TPI conformation via hydrogen and hydrophobic interactions with loop 6. Point mutations in GlTPI-C222 and the equivalent cysteine residue in the cytosolic TPI from Arabidopsis reduced the enzymatic activity of those enzymes [69, 70]. Point mutations in SmTPI-C221 partially abrogate catalysis, having a reduction of approximately 10-fold in catalytic efficiency. This reduction in enzymatic activity correlates with a deficiency in complementation of an *E*. *coli* strain devoid of *tpi* by plasmids harboring mutations in residue SmTPI-C221 **(S4 Fig)**. In sum, this work provides a blueprint of TPIs from parasitic helminths as a target for schistosomiasis and cysticercosis vaccine or drug development.

## Supporting information

S1 Fig Purification of heterologous expressed TsTPI and SmTPI. 10% SDS-PAGE showing the purified TPsI. The molecular mass of each protein is approximately 25 kDa.
(TIF)

S2 Fig Prediction of linear epitopes in HsTPI. The amino acid corresponding to the linear epitopes are depicted by their start and end. The probability score and the color code of epitope is indicated. The localization of individual epitopes is present only in molecule A
(TIF)

S3 Fig Mutations in residue SmTPI-C221 decrease TPI activity. **A)** Chemical structure of possible modification in reactive cysteines and their amino acid mimicry **B)** Complementation assay by bacterial strains harboring SmTPI-C221 point mutants that resemble its oxidation of thiol conjugation.
(TIF)

S4 Fig Gel filtration elution profile of wild-type and point mutants of SmTPI. The elution profile of all proteins corresponds to a dimer of approximately 50 kDa.
(TIF)

S5 Fig Structural rational for the thermal stability exhibited by SmTPI. **A**) Amino acid sequence alignment between TsTPI and SmTPI. Both protein share 60% amino acid identity and the main differences are in α1, β2, and α3 and the C-terminal part of α6. **B** and **C**) Ribbon and surface representation of SmTPI and TsTPI showing the stabilizing interactions present in α1, β2, and α3 of SmTPI.
(TIF)

S1 Table Optimized nucleotide coding sequences of TsTPI and SmTPI for its heterologous expression in *E. coli*. (DOCX)

S2 Table Data collection and refinement statistics. (DOCX)
