# Supplementary figures and images for: Crystal structures of Triosephosphate Isomerases from Taenia solium and Schistosoma mansoni provide insights for vaccine rationale and drug design against helminth parasites
Source: PLoS Negl Trop Dis. 2020 Jan 10;14(1):e0007815. doi: 10.1371/journal.pntd.0007815 (PMC6980832; doi:10.1371/journal.pntd.0007815)

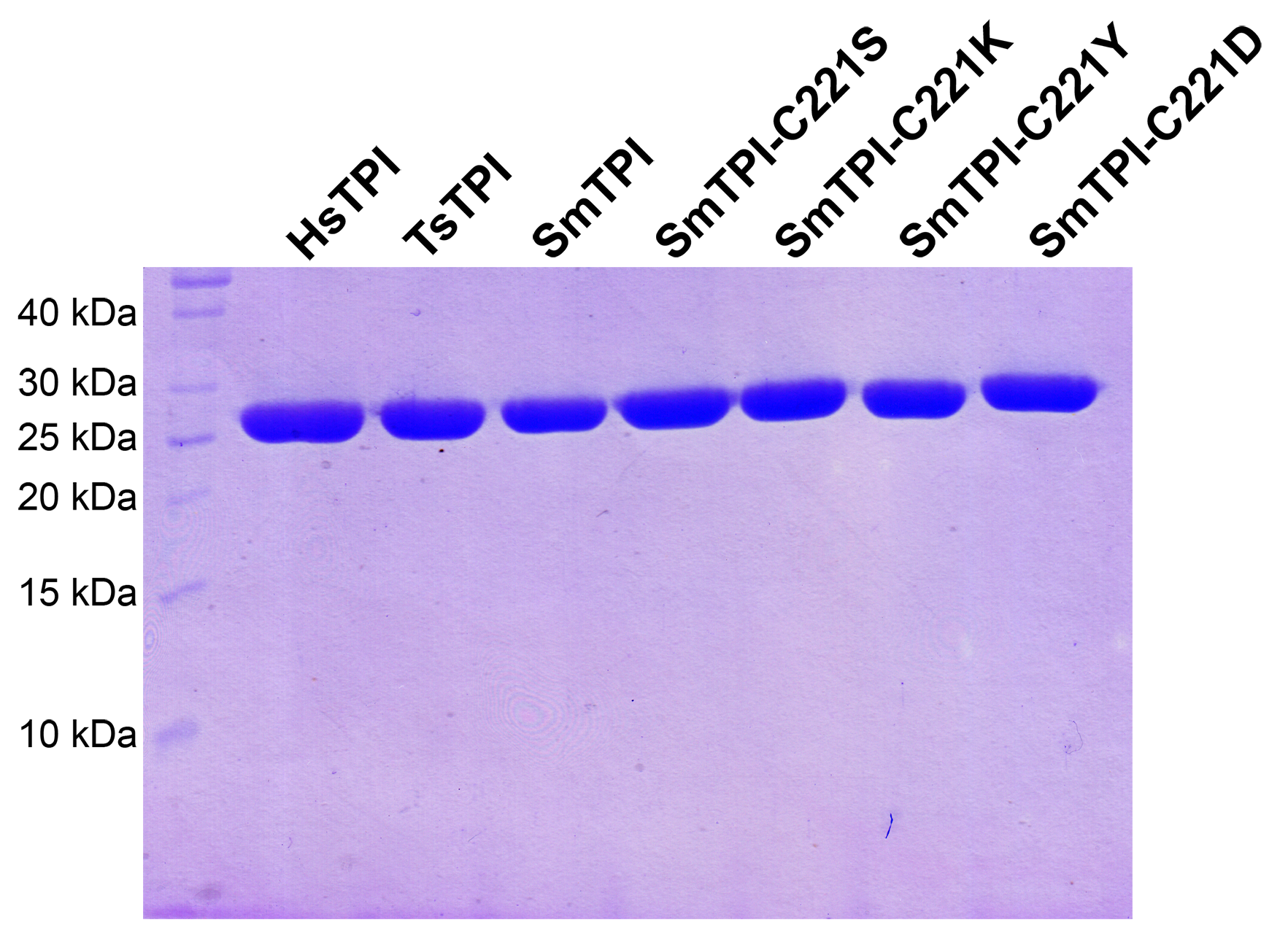

Supplement: S1 Fig — 10% SDS-PAGE showing the purified TPsI. The molecular mass of each protein is approximately 25 kDa. (TIF) [file pntd.0007815.s001.tif]

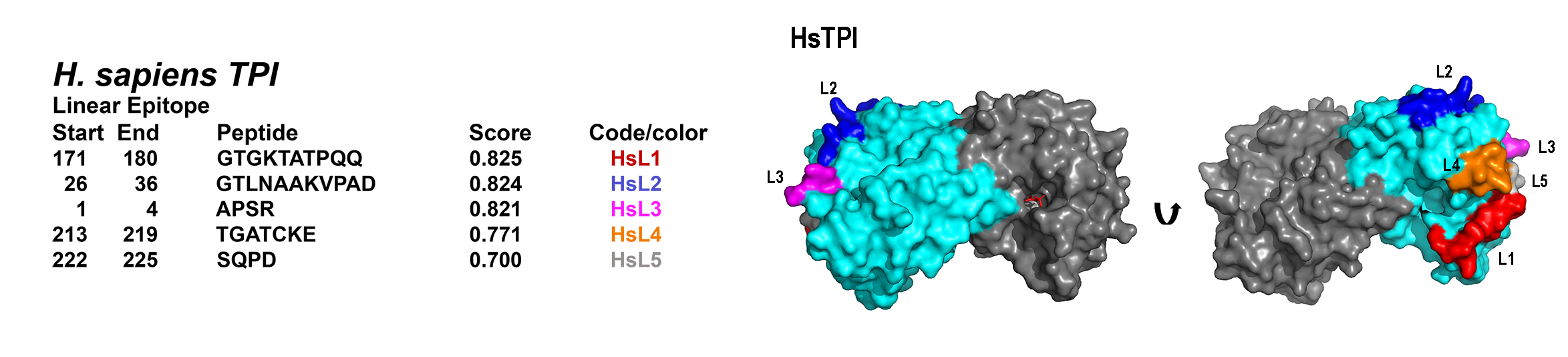

Supplement: S2 Fig — The amino acid corresponding to the linear epitopes are depicted by their start and end. The probability score and the color code of epitope is indicated. The localization of individual epitopes is present only in molecule A (TIF) [file pntd.0007815.s002.tif]

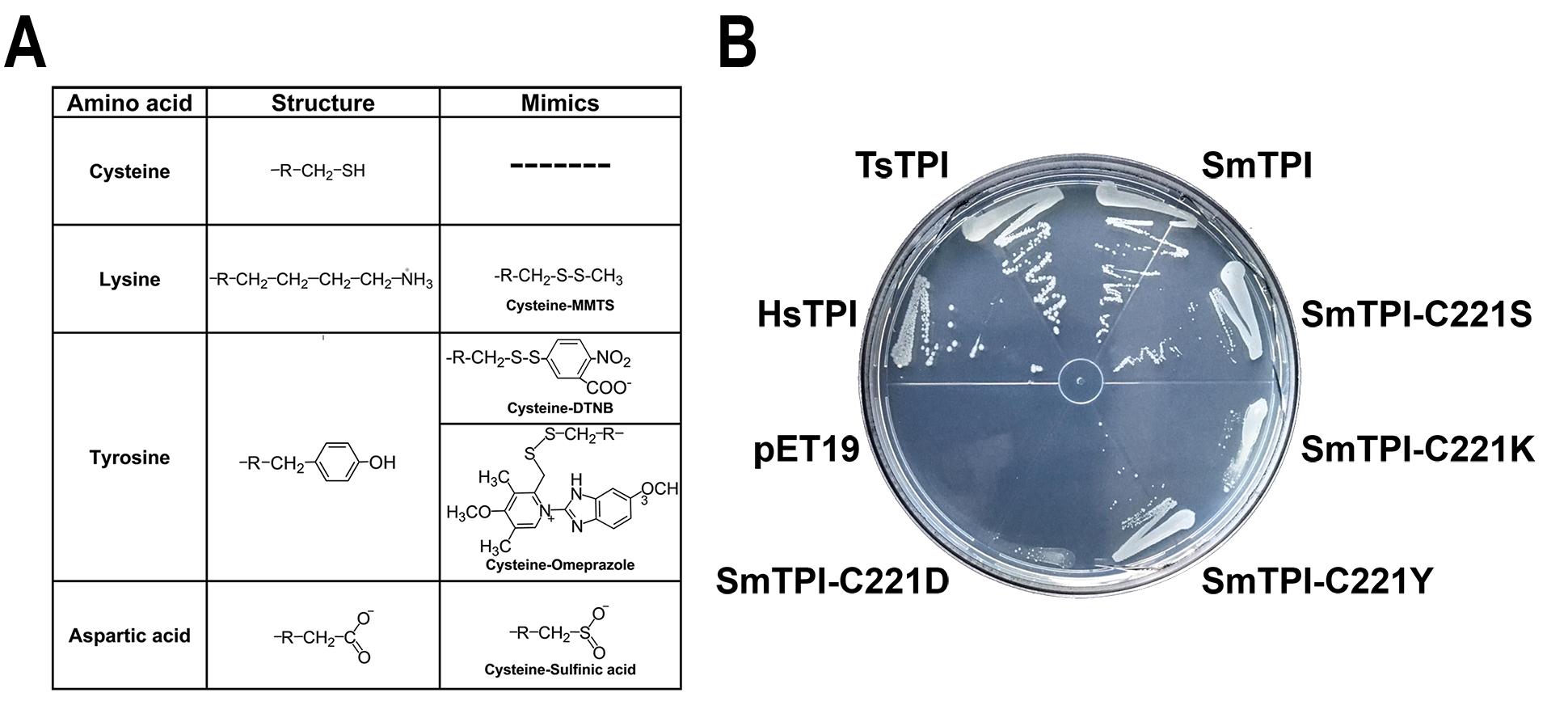

Supplement: S3 Fig — A) Chemical structure of possible modification in reactive cysteines and their amino acid mimicry B) Complementation assay by bacterial strains harboring SmTPI-C221 point mutants that resemble its oxidation of thiol conjugation. (TIF) [file pntd.0007815.s003.tif]

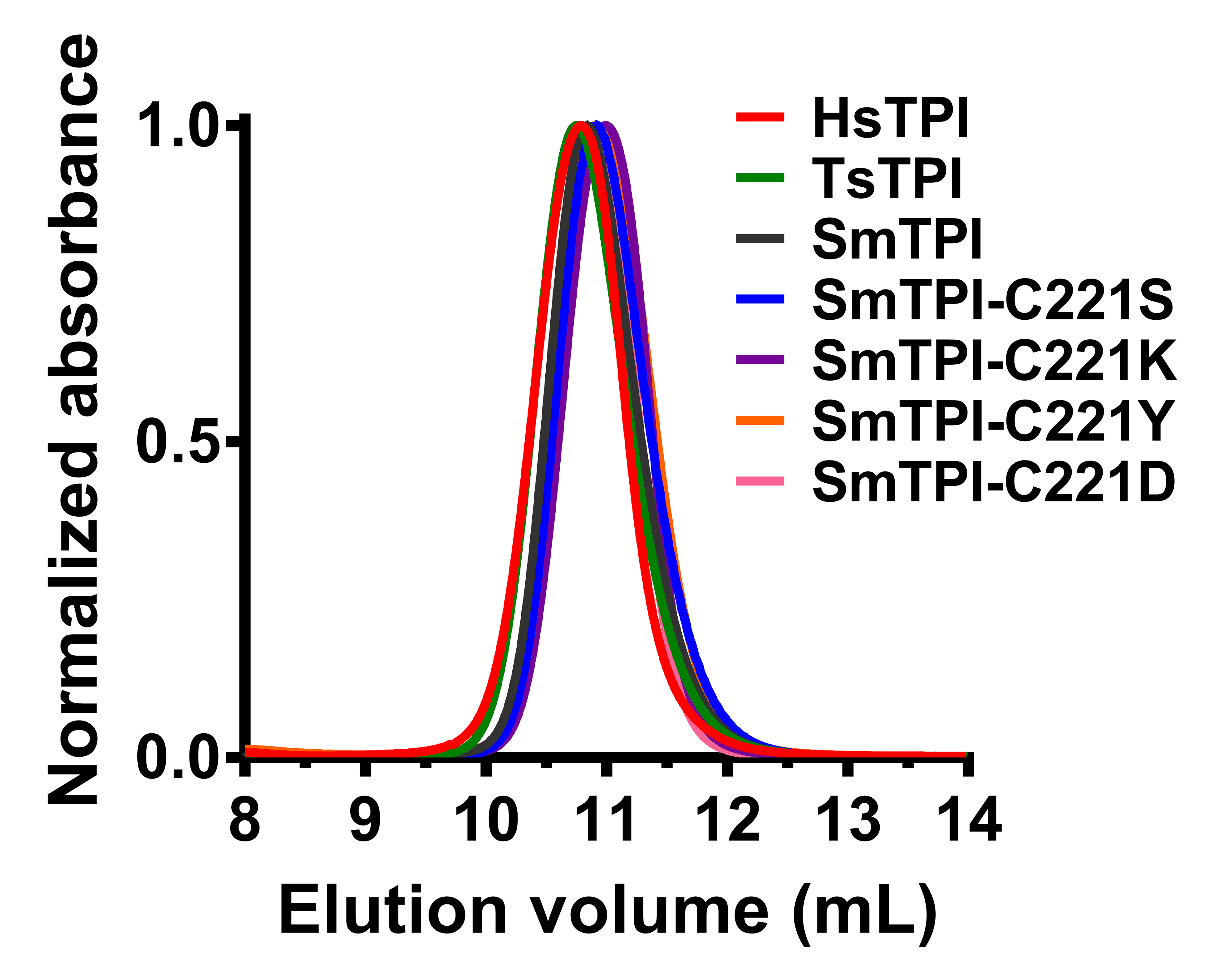

Supplement: S4 Fig — The elution profile of all proteins corresponds to a dimer of approximately 50 kDa. (TIF) [file pntd.0007815.s004.tif]

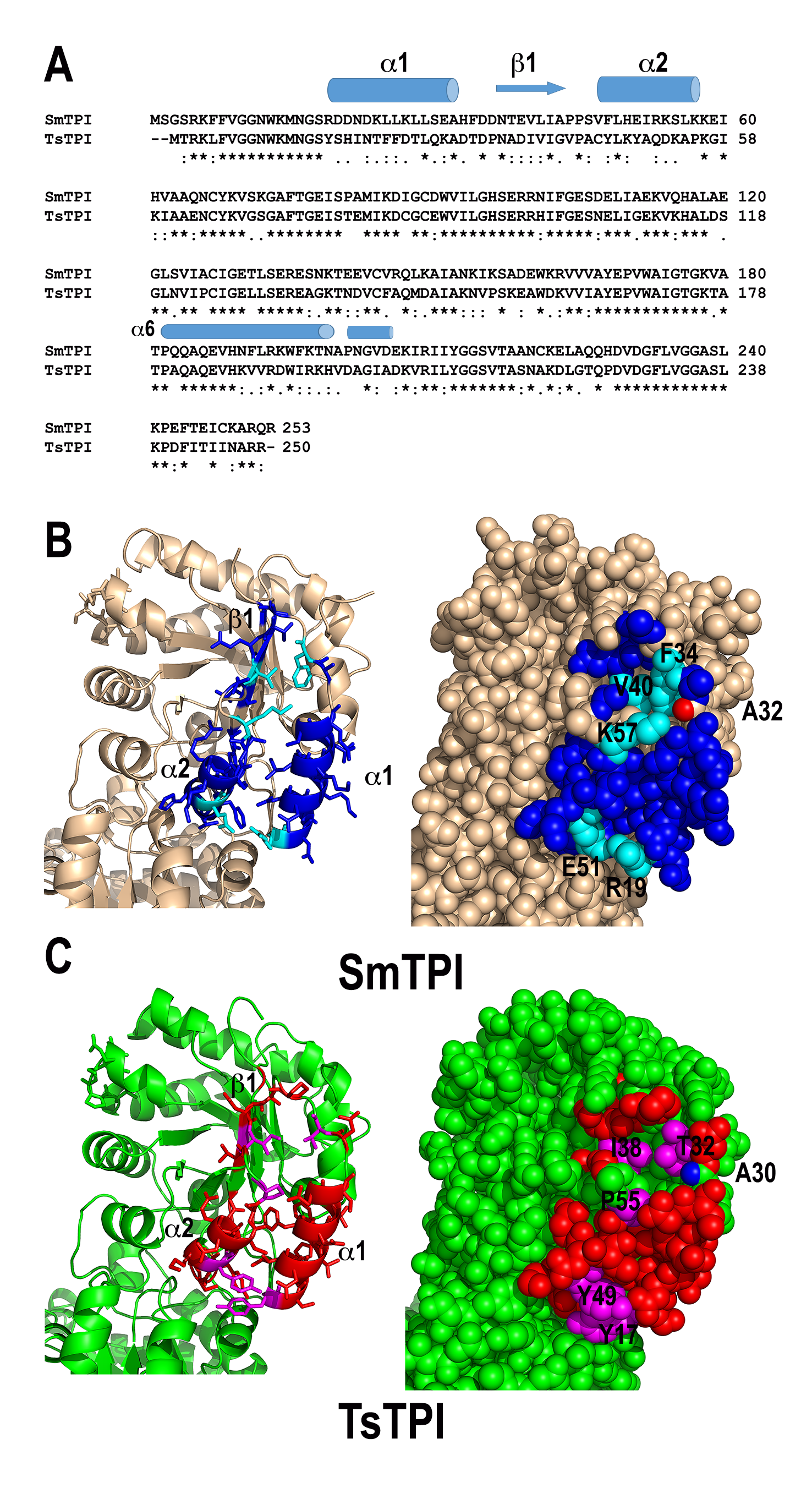

Supplement: S5 Fig — A) Amino acid sequence alignment between TsTPI and SmTPI. Both protein share 60% amino acid identity and the main differences are in α1, β2, and α3 and the C-terminal part of α6. B and C) Ribbon and surface representation of SmTPI and TsTPI showing the stabilizing interactions present in α1, β2, and α3 of SmTPI. (TIF) [file pntd.0007815.s005.tif]
