# Supplementary material for: Crystal structures of Triosephosphate Isomerases from Taenia solium and Schistosoma mansoni provide insights for vaccine rationale and drug design against helminth parasites
Source: PLoS Negl Trop Dis. 2020 Jan 10;14(1):e0007815. doi: 10.1371/journal.pntd.0007815 (PMC6980832; doi:10.1371/journal.pntd.0007815)
Supplement: S1 Table — (DOCX) [file pntd.0007815.s006.docx]

**Table 1S Optimized nucleotide coding sequences of TsTPI and SmTPI for its heterologous expression in *E. coli***

SmTPI

ATGTCTGGTTCTCGTAAGTTTTTTGTGGGAGGAAATTGGAAAATGAATGGTTCCCGCGATGACAATGATAAATTGTTAAAACTTCTGTCTGAAGCCCATTTTGACGACAACACTGAGGTCTTGATTGCCCCCCCCTCCGTGTTTTTGCATGAGATTCGCAAGTCGCTGAAGAAGGAAATCCACGTCGCCGCACAGAACTGCTATAAAGTGTCTAAGGGTGCCTTCACGGGCGAAATCTCTCCGGCTATGATTCGCGATATTGGTTGTGACTGGGTTATTCTGGGGCACTCCGAGCGTCGTAACATCTTTGGAGAATCTGACGAGCTTATCGCGGAGAAAGTCCAACACGCCCTGGCCGAGGGCCTGTCGGTCATTGCCTGCATTGGAGAGACGCTGTCGGAACGCGAATCTAATAAGACGGAGGAAGTTTGCGTCCGCCAGCTTAAAGCTATTGCTAATAAGATCAAAAGTGCCGATGAGTGGAAGCGTGTAGTTGTAGCGTATGAACCTGTGTGGGCGATTGGCACTGGAAAAGTGGCAACGCCACAACAAGCACAAGAGGTTCATAACTTCTTACGCAAGTGGTTTAAAACTAATGCACCGAATGGAGTGGACGAGAAGATTCGTATCATTTATGGCGGAAGTGTAACAGCGGCCAATTGCAAGGAGCTGGCTCAACAACATGATGTGGATGGCTTTTTGGTCGGTGGGGCATCCCTGAAGCCGGAGTTTACTGAAATTTGTAAAGCGCGCCAGCGCTAAGGATCCGGCTGCTAACAAAGCCCGAAAGGAAGCTGAGTTGGCTGCTGCCACCGCTGAGCAATAACTAGCATAACCCCTTGGGGCTCTAAACGGGTCTGAGGGGTTTTTGCTGAAAGGAGGACTATATCCGGATTATCCCGCAGAGGCCCGGCAGTACGGCATACAGCTATGCTACAGCATCCAGGTGACGTGCGAGATGACGATGAGCGCATTGTAGATTCCATACCGGTGCTGACTGCGTAGCATTACTGGATTACTACGCATAAGCTACGAGATAGCTGCACATGAATCCTGAAGACAAGGCCTTCGGTGATACGCCCATAT

TsTPI

ATGACCCGTAAATTGTTCGTGGGCGGGAATTGGAAGATGAACGGGTCTTATTCCCACATCAACACGTTTTTCGATACGTTGCAGAAGGCCGACACGGACCCTAATGCTGACATCGTGATCGGTGTTCCGGCTTGCTACTTGAAATATGCCCAAGATAAGGCCCCTAAGGGAATCAAGATTGCCGCTGAAAACTGTTATAAAGTAGGCTCCGGGGCCTTCACCGGCGAGATTAGTACTGAGATGATTAAAGACTGTGGCTGTGAATGGGTGATTCTGGGGCACTCAGAGCGCCGTCACATTTTCGGGGAATCGAACGAGTTGATCGGTGAGAAGGTGAAACATGCCCTGGACAGTGGGCTTAATGTTATCCCCTGTATCGGAGAGTTGTTATCAGAACGTGAAGCGGGGAAGACAAACGATGTCTGTTTCGCACAAATGGACGCAATCGCCAAGAATGTGCCGTCAAAAGAAGCCTGGGACAAGGTAGTGATCGCGTATGAGCCCGTGTGGGCGATCGGAACAGGCAAGACTGCGACGCCCGCTCAAGCCCAGGAAGTACATAAAGTAGTACGTGACTGGATTCGCAAGCACGTAGATGCCGGTATTGCCGATAAAGTTCGTATTCTGTACGGAGGGTCCGTCACTGCTTCAAATGCCAAAGATTTAGGGACCCAACCTGATGTGGACGGTTTTTTGGTAGGCGGTGCTTCATTAAAACCGGACTTCATTACGATTATTAATGCTCGTCGCTAAGGATCCGGGCTGCTAACAAAGCCCGAAAGGAAGCTGAGTTGGCTGCTGCACCGCTGAGCAATACTAGCATAACCCCTTGGGGCTCTAACGGGTCTTGAGGGGTTTTTGCTGAAGGAGGAACTATATCCGGATATCCCGCAAGAGGCCGGGCAGTACCGGCATACAGTCTATGCTAACAGCATCAGGGTGACGGTGCGAGATGACGATGAGCGCATGGTAGATTTCATACACGGTGCTGACTGGCTAGCAATAACTGGATAACTTACCGCATAAGCTATCGTAGTAGCGTCACTGAGAATTCTGGAGAACAGAAAGGGGCCCTCTG
