# Supplementary material for: Crystal structures of Triosephosphate Isomerases from Taenia solium and Schistosoma mansoni provide insights for vaccine rationale and drug design against helminth parasites
Source: PLoS Negl Trop Dis. 2020 Jan 10;14(1):e0007815. doi: 10.1371/journal.pntd.0007815 (PMC6980832; doi:10.1371/journal.pntd.0007815)
Supplement: S2 Table — (DOCX) [file pntd.0007815.s007.docx]

|  | TsTPI | SmTPI |
| --- | --- | --- |
| **PDB code ID** | **6OOG** | **6OOI** |
| *Data* *processing* |  |  |
| Space group | P 41 21 2 | P 43 |
| a, b, c (Å) | 66.4 66.4 155.6 | 108.1 108.1 182.7 |
| α, β, γ (°) | 90 90 90 | 90 90 90 |
| Resolution (Å) | 66.04 – 2.02 | 58.61 – 2.14 |
| R_merge_ | 0.287 (2.307) | 0.133 (0.688) |
| I/σI | 7.2 (1.2) | 5.6 (1.6) |
| CC_1/2_ | 0.992 (0.339) | 0.984 (0.639) |
| Completeness (%) | 99 (99) | 100 (100) |
| Redundancy | 10.3 (9.8) | 4.5 (4.2) |
|  |  |  |
| *Refnement* |  |  |
| Resolution (Å) | 61.07 – 2.11 | 58.63 – 2.19 |
|  | (2.11 – 2.02) | (2.19 – 2.14) |
| No. of reflections | 23529 / 1176 * | 114956 / 2014* |
| Rwork / Rfree (%) | 18.4 / 22.4 | 18.1 / 22.9 |
| Number of non-hydrogen atoms | 2091 | 16313 |
| macromolecules |  |  |
| ligands | 10 | 98 |
| solvent | 189 | 1075 |
| RMS(bonds) | 0.007 | 0.008 |
| RMS(angles) | 0.82 | 0.83 |
| Ramachandran favored (%) | 97.2 | 97.3 |
| Ramachandran allowed (%) | 2.80 | 2.72 |
| Ramachandran outliers (%) | 0.0 | 0.0 |
| Average B-factor | 25.66 | 24.12 |

**Table S2:** **Data processing and refinement**

Values in parentheses are for highest-resolution shell. * Corresponds to the fraction of reflections of the *R_free_* set
